# Effect of Long-Term Supplementation with Acetic Acid on the Skeletal Muscle of Aging Sprague Dawley Rats

**DOI:** 10.3390/ijms23094691

**Published:** 2022-04-23

**Authors:** Hitomi Maruta, Reina Abe, Hiromi Yamashita

**Affiliations:** 1 Department of Nutritional Science, Faculty of Health and Welfare Science, Okayama Prefectural University, 111 Kuboki, Soja 719-1197, Okayama, Japan; maruta@fhw.oka-pu.ac.jp; 2 Graduate School of Health and Welfare Science, Okayama Prefectural University, 111 Kuboki, Soja 719-1197, Okayama, Japan; cras-amet.sper0@docomo.ne.jp

**Keywords:** skeletal muscle, mitochondria, type I fiber, acetic acid, AMPK, aging

## Abstract

Mitochondrial function in skeletal muscle, which plays an essential role in oxidative capacity and physical activity, declines with aging. Acetic acid activates AMP-activated protein kinase (AMPK), which plays a key role in the regulation of whole-body energy by phosphorylating key metabolic enzymes in both biosynthetic and oxidative pathways and stimulates gene expression associated with slow-twitch fibers and mitochondria in skeletal muscle cells. In this study, we investigate whether long-term supplementation with acetic acid improves age-related changes in the skeletal muscle of aging rats in association with the activation of AMPK. Male Sprague Dawley (SD) rats were administered acetic acid orally from 37 to 56 weeks of age. Long-term supplementation with acetic acid decreased the expression of atrophy-related genes, such as atrogin-1, muscle RING-finger protein-1 (MuRF1), and transforming growth factor beta (TGF-β), activated AMPK, and affected the proliferation of mitochondria and type I fiber-related molecules in muscles. The findings suggest that acetic acid exhibits an anti-aging function in the skeletal muscles of aging rats.

## 1. Introduction

With a growing aging population, the prevalence of chronic diseases is increasing. Aging is accompanied by the loss of skeletal muscle mass and function (referred to as sarcopenia), with severe consequences on quality of life. Aged muscle is characterized by a decline in both the number and size of muscle fibers, and contributes to a decline in metabolic, respiratory, and exercise functions [1,2]. The bioenergetics of skeletal muscle largely rely on the metabolic flexibility of its mitochondria [3,4]. Mitochondrial function in skeletal muscles, which plays an essential role in maintaining health throughout life, declines during aging concomitant with decreased exercise and physical activity [5,6,7,8]. Conley et al. [9] reported a 50% reduction in oxidative capacity in older human subjects compared to that observed in young subjects due to reduced mitochondrial volume and function. A substantial decrease in mitochondrial oxidative capacity in aging muscles contributes to a reduced exercise capacity in elderly people [10] and an increased lipid accumulation [11,12]. In old age, mitochondria evidently exhibit a lower density and reduced content of mitochondrial DNA [13,14]. Age-related impaired mitochondrial function in muscles is attributed to a reduced mitochondrial DNA content, which leads to functional changes in the elderly [8]. These findings suggest that aging is associated with the dysregulation of energy metabolism and, at least in part, with mitochondrial dysfunction. Protection against reduction in mitochondrial function would improve age-related changes in skeletal muscle function.

Previously, we reported that orally administered acetic acid contributes to enhanced O_2_ consumption and lipid oxidation with concomitant increases in the AMP/ATP ratio and phosphorylation of AMP-activated protein kinase (AMPK) in obesity-linked type 2 diabetic Otsuka Long-Evans Tokushima Fatty (OLETF) rats [15]. Acetic acid activates AMPK, induces gene and protein expression of myoglobin and GLUT4, stimulates glucose incorporation, and suppresses lipid accumulation in L6 cells [16]. Furthermore, acetic acid activates the G protein-coupled receptor 43 (GPR43) and AMPK in L6 cells and also stimulates the gene expression associated with slow-twitch fibers and mitochondria by regulating nuclear factors, such as the nuclear factor of activated T cells 1 (NFATc1), myocyte enhancer factor 2A (MEF2A), peroxisome proliferator-activated receptor-gamma coactivator 1 alpha (PGC-1α), and calcium/calmodulin-dependent protein kinase kinase (CaMKK) through calcium signaling [17].

AMPK is a heterotrimeric protein kinase that plays a key role in the regulation of whole-body energy by phosphorylating key metabolic enzymes in both biosynthetic and oxidative pathways [18,19]. Various cellular or metabolic stressors that either inhibit ATP synthesis (e.g., heat shock, hypoxia, or glucose starvation) or enhance ATP consumption (such as physical exercise) increase the intracellular AMP/ATP ratio, leading to AMPK activation. A well-known function of AMPK is the inactivation of acetyl-CoA carboxylase (ACC), the rate-limiting enzyme in fatty acid synthesis [20,21]. Activated AMPK phosphorylates ACC, which leads to a decrease in intracellular malonyl-CoA, blocks fatty acid synthesis, activates carnitine palmitoyl-CoA transferase I, increases fatty acid oxidation to generate energy, and enhances energy expenditure. In addition, activated AMPK phosphorylates PGC-1α [22], which plays a key role in the regulation of mitochondrial biogenesis and oxidative metabolism. AMPK activates the NAD^+^-dependent type III deacetylase sirtuin 1 (SIRT1) by increasing the intracellular NAD^+^/NADH ratio via accelerated NAD^+^ synthesis, leading to the deacetylation and activation of PGC-1α [23]. In skeletal muscle, the activation of the AMPK-SIRT1-PGC-1α axis resulting from either muscle contraction or chemical stimulation leads to an increase in the levels of slow-twitch muscle fibers via an enhancement of mitochondrial biogenesis, oxidative metabolism, and type I myofiber synthesis [6,22]. AMPK is reportedly involved in the regulation of exercise performance [24,25,26] and anti-aging effects [27]. As described above, acetic acid treatment stimulates fatty acid metabolism and glucose incorporation in L6 skeletal muscle cells through AMPK activation. Furthermore, the activation of AMPK by acetic acid is associated with the activation of the G-protein coupled receptor GPR43, which is expressed in skeletal muscles as well as adipose tissues, intestine, and immune cells, and is involved in the suppression of lipid accumulation and maintenance of body energy homeostasis [28,29,30,31,32], leading to an increase in gene expression related to slow-twitch fibers in L6 cells [17].

We hypothesized that long-term acetic acid supplementation may improve age-related changes in skeletal muscle function in aging rats via the AMPK and GPR43 pathways. In this study, we investigated the effects of long-term acetic acid supplementation on the skeletal muscles of aging Sprague Dawley (SD) rats.

## 2. Results

### 2.1. Effect of Acetic Acid on Age-Related Changes in Energy Metabolism

**Figure 1:**
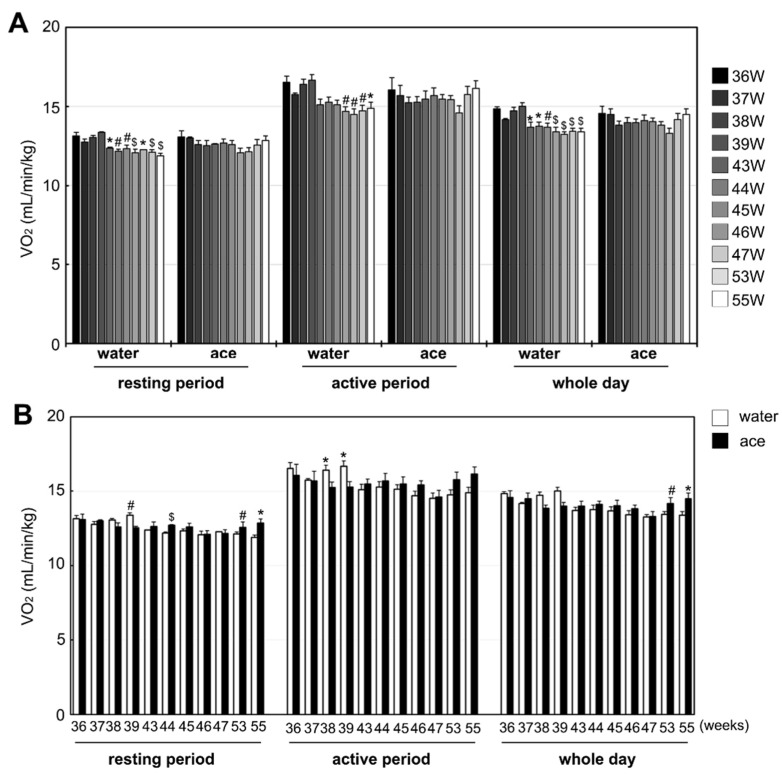
Oxygen consumption (VO_2_) of Sprague Dawley (SD) rats in the water and acetic acid (ace) groups from 36 to 55 weeks of age (**A**), and comparison between VO_2_ of SD rats in the water and ace groups (**B**) during the resting, active, and whole-day periods. The resting period was 10:00 am to 8:00 pm, the active period was 8:00 pm to 8:00 am, and the whole-day period was from 10:00 am to 8:00 am. (**A**): Results were analyzed with one-way ANOVA followed by the Tukey–Kramer post hoc test for multiple comparisons. * *p* < 0.05, ^#^ *p* < 0.01, ^$^ *p* < 0.001, statistically significant versus the value obtained at 36 weeks of age. (**B**): Results were analyzed with Student’s *t*-test. * *p* < 0.05, ^#^ *p* < 0.01, ^$^ *p* < 0.001, statistically significant versus the value of water group. Values shown represent means ± SE for 4–5 rats.

Body weight and total food intake were not significantly different between the acetic acid group and the water group at 56 weeks of age, as described in the Materials and Methods Section. To determine the effect of acetic acid on age-related changes in respiratory metabolism, oxygen consumption (VO_2_) was measured at each week of age, from 36 to 55 weeks (Figure 1A). VO_2_ in the water group significantly decreased from 43 to 55 weeks compared with the observation in rats aged 36 weeks, during resting and whole-day periods. The water intake of rats aged 46 weeks was significantly lower than that of rats aged 36 weeks during the active period. VO_2_ did not significantly change in the acetic acid group until 55 weeks of age. The acetic acid group showed higher VO_2_ than the water group at the ages of 54 and 55 weeks during the entire day period (Figure 1B).

### 2.2. Effect of Acetic Acid on the Fiber Types of Muscles

**Figure 2:**
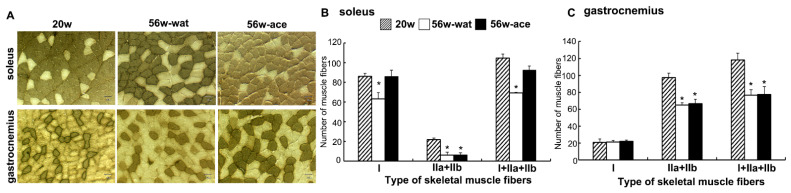
Light micrographs of the soleus and gastrocnemius muscles of rats at 20 and 56 weeks of age stained for ATPase (**A**). Number of fibers in the soleus (**B**) and gastrocnemius muscles (**C**). 20w: SD rats at 20 weeks of age; 56w-wat: SD rats at 56 weeks of age in the water group, 56w-ace: SD rats at 56 weeks of age in the acetic acid group. Fiber number was counted in a 0.45 mm^2^ area. Scale bar: 50 μm. Values shown represent means ± SE for 4–5 rats. Results were analyzed with one-way ANOVA followed by the Tukey–Kramer post hoc test for multiple comparisons. * *p* < 0.05, statistically significant versus value obtained at 20 weeks of age.

To examine the effect of acetic acid on the numbers of each muscle fiber type during the aging process, the soleus and gastrocnemius muscles were analyzed by ATPase staining. The number of type I and type II fibers was significantly decreased in the soleus at 56 weeks of age compared to that at 20 weeks of age (Figure 2). Treatment with acetic acid protected against a decrease in the number of type I fibers in the soleus muscles of rats at 56 weeks of age. The number of type I fibers did not change, whereas that of type II fibers was significantly decreased in the gastrocnemius muscle in both the water and acetic acid groups (Figure 2).

### 2.3. Effect of Acetic Acid on the Gene Expression of Skeletal Muscles

**Figure 3:**
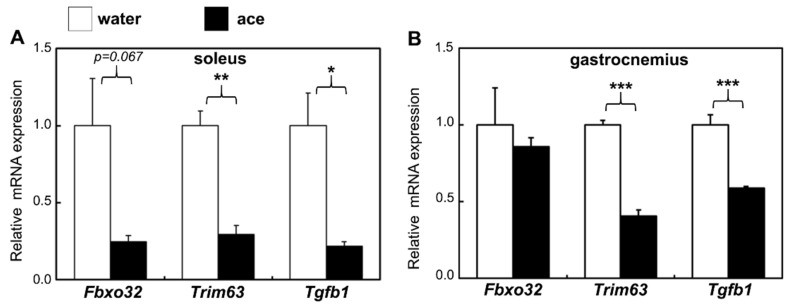
Effect of long-term supplementation with acetic acid on the relative mRNA expression of Fbxo32, Trim63, and Tgfb1 genes in the soleus (**A**) and gastrocnemius (**B**) muscles of SD rats at 56 weeks of age in the water (□) and acetic acid (ace, ■) groups. Values represent means ± SE for 4–5 rats. * *p* < 0.05, ** *p* < 0.01, *** *p* < 0.001, statistically significant versus the value of water group. Results were analyzed with Student’s *t*-test.

**Figure 4:**
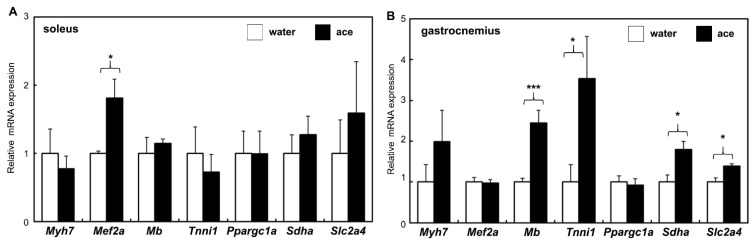
Effect of long-term supplementation of acetic acid on the relative mRNA expression of slow twitch fiber-related genes in the soleus (**A**) and gastrocnemius (**B**) muscles of SD rats at 56 weeks of age in the water (□) and acetic acid (ace, ■) groups. Values represent means ± SE for 4–5 rats. * *p* < 0.05, *** *p* < 0.001, statistically significant versus the value of water group. Results were analyzed with Student’s *t*-test.

To determine the effects of aging and treatment with acetic acid on muscle atrophy and fibrosis, the expression levels of muscle atrophy genes, including those encoding atrogin-1(*Fbxo32*), muscle RING-finger protein-1 (MuRF1, *Trim63*), and transforming growth factor beta (TGF-β, *Tgfb1*), were analyzed. The expression levels of these genes were significantly decreased in the soleus muscles of rats at 56 weeks of age in the acetic acid group compared with the expression levels observed in the water group (Figure 3A). MuRF1 and TGF-β gene expression levels were significantly decreased in the gastrocnemius muscles of rats in the acetic acid group compared to those in the water group (Figure 3B). The expression of the muscle transcription factor MEF2A gene was significantly increased in the soleus muscles of rats treated with acetic acid compared with that in the soleus muscles of the rats in the water group. The expression of myoglobin (*Mb*), tropomyosin (*Tnni1*), succinate dehydrogenase (SDH, *Sdha*), and GLUT4 (*Slc2a4*) genes was also significantly increased in the gastrocnemius muscles of the rats in the acetic acid group at 56 weeks of age compared to that observed in the gastrocnemius muscles of rats in the water group (Figure 4).

### 2.4. Long-Term Supplementation with Acetic Acid Stimulates the Nuclear Localization of MEF2A and PGC-1α in the Soleus Muscle of Rats during Aging

**Figure 5:**
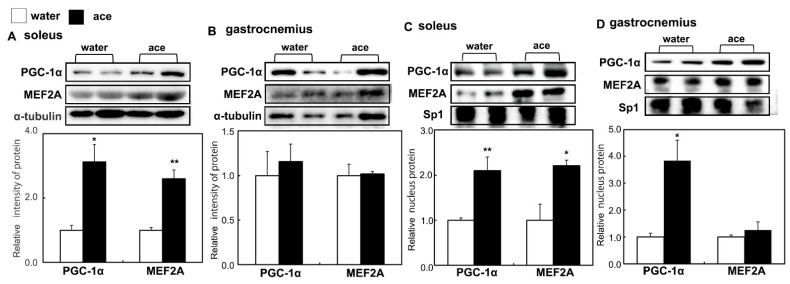
Effects of long-term supplementation of acetic acid on the expression of peroxisome proliferator-activated receptor-gamma coactivator 1 alpha (PGC-1α) and myocyte enhancer factor 2A (MEF2A) proteins in the soleus (**A**) and gastrocnemius (**B**) muscles and the nuclear localization of PGC-1α and MEF2A in the soleus (**C**) and gastrocnemius (**D**) muscles of SD rats at 56 weeks of age in the water and acetic acid (ace) groups. Values represent means ± SE for 4–5 rats. * *p* < 0.05, ** *p* < 0.01, statistically significant versus the value of water group. Results were analyzed with Student’s *t*-test.

MEF2A and the PGC-1α transcription co-factor are associated with the proliferation and generation of slow-twitch muscle fiber proteins and are localized in the nucleus for transcriptional activity [22,33,34]. We analyzed the nuclear localization of MEF2A and PGC-1α following long-term supplementation with acetic acid. MEF2A and PGC-1α proteins were increased in the soleus muscle and in the nuclei of the soleus muscle cells of rats with a long-term supplementation of acetic acid as compared with their expression in the soleus muscles of the rats in the water group (Figure 5A,C). In the gastrocnemius muscle, the expression of these two proteins did not change, while PGC-1α expression was increased in the nucleus of the acetic acid group compared with the water group (Figure 5B,D).

### 2.5. Mitochondrial DNA (mtDNA) Analysis and SDH Staining

**Figure 6:**
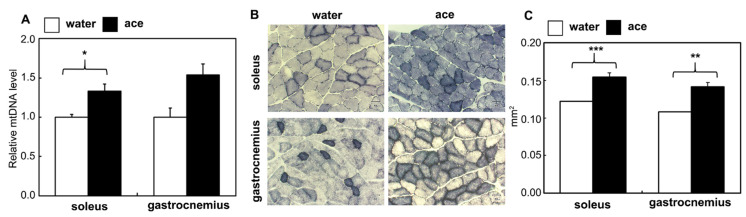
Effect of long-term supplementation of acetic acid on mitochondrial DNA (mtDNA) content (**A**), succinate dehydrogenase (SDH) staining (**B**), and Image J analysis of SDH staining (**C**) in the soleus and gastrocnemius muscles of SD rats at 56 weeks of age. A: Values represent means ± SE for 4–5 rats. * *p* < 0.05, ** *p* < 0.01, *** *p* < 0.001, statistically significant versus the value of water group. Results were analyzed with Student’s *t*-test.

To examine the effect of acetic acid supplementation on mitochondrial proliferation, the relative quantity of mtDNA and the staining level of SDH in muscle sections were analyzed. Significantly higher quantities of mtDNA were evident in the soleus muscle of the acetic acid group than in that of the water group (Figure 6A). The SDH staining level was higher in the soleus and gastrocnemius muscles of the acetic acid group than in those of the water group (Figure 6B,C).

### 2.6. Effect of Acetic Acid on GPR43 Gene Expression in the Skeletal Muscles of Aging Rats

**Figure 7:**
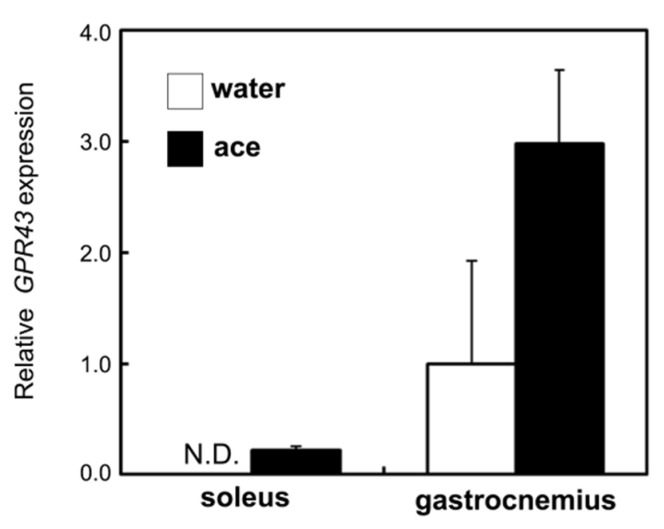
Long-term supplementation with acetic acid tends to induce the expression of G protein-coupled receptor 43 (GPR43) gene in the soleus muscle of SD rats at 56 weeks of age. Values represent means ± SE for 4–5 rats. N.D.: not detected.

GPR43 is expressed in a large variety of tissues, including skeletal muscles [17], and it is involved in both mitochondrial proliferation and the inhibition of fat accumulation. GPR43 plays a key role in the regulation of lifestyle-related diseases [17,28]. The expression of GPR43 was not detected in the soleus muscles of rats at 56 weeks of age in the water group. However, supplementation with acetic acid induced and improved GPR43 expression in the soleus muscle. With respect to the gastrocnemius muscle, the expression also tended to be increased in rats in the acetic acid group compared with those in the water group (Figure 7).

### 2.7. Supplementation with Acetic Acid Protects Intramuscular Lipid Accumulation in the Soleus Muscle

**Figure 8:**
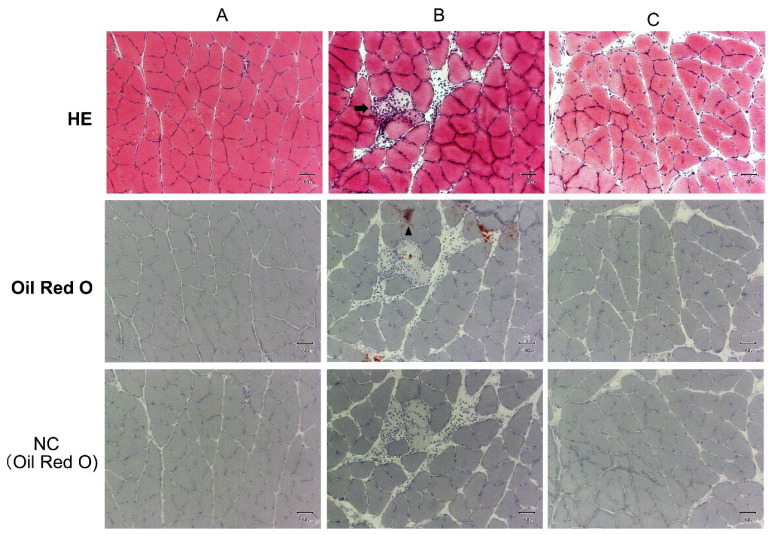
Long-term supplementation of acetic acid attenuates lipid accumulation and fibrosis in the soleus muscles of SD rats at 56 weeks of age. (**A**) SD rats at 20 weeks of age; (**B**) SD rats of the water group at 56 weeks of age; (**C**) SD rats of the acetic acid group at 56 weeks of age. HE: hematoxylin and eosin stain; Oil red O: Oil red O stain; NC: Before Oil red O stain; subjected to ethanol treatment with the subsequent removal of lipids. Arrowhead: lipid accumulation; arrow: necrosis. Scale bar: 50 μm.

During aging, lipids accumulate within the skeletal muscle and have a higher triglyceride content in the aged muscle, heart, and liver tissues [11,35]. Histological analyses indicated that the soleus muscles of the rats of the water group had increased intramuscular lipid accumulation and showed necrosis at 56 weeks of age as compared with the observations at 20 weeks of age. The changes did not occur when supplemented by acetic acid (Figure 8).

### 2.8. Activation of AMPK and Akt Pathway

**Figure 9:**
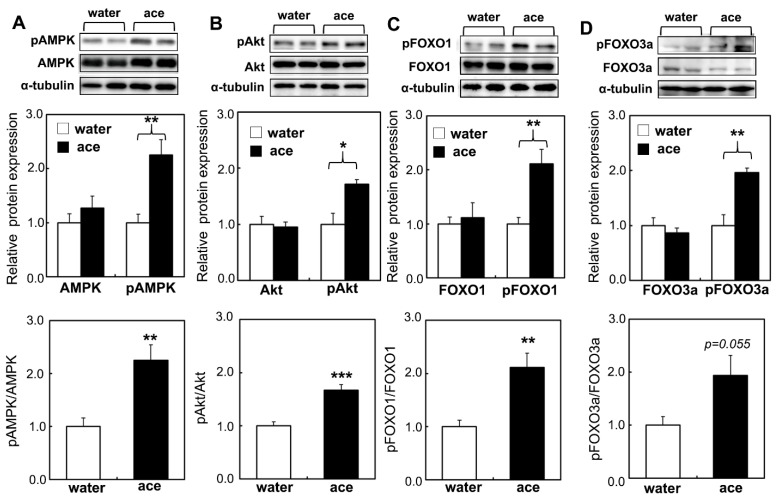
Long-term supplementation of acetic acid increases the phosphorylation of AMP-activated protein kinase (AMPK), Akt, forkhead box protein O (FOXO)1, and FOXO3a. AMPK and pAMPK (**A**), Akt and pAkt (**B**), FOXO1 and pFOXO1 (**C**), FOXO3a and pFOXO3a (**D**), and α-tubulin were analyzed in the soleus muscle of water and acetic acid groups. They were analyzed by Western blotting, as described in the Materials and Methods Section. Values represent means ± SE for 4–5 rats. * *p* < 0.05, ** *p* < 0.01, *** *p* < 0.001, statistically significant versus the value of water group. Results were analyzed with Student’s *t*-test.

Lipid deposition in the skeletal muscle may be due to the alteration of molecules associated with energy metabolism during aging. To examine the effect of long-term acetic acid supplementation on the modulation of molecules related to energy metabolism in the skeletal muscle, the phosphorylation of AMPK in the soleus muscle was examined. The levels of phosphorylated AMPK in the soleus muscle were significantly higher in the acetic acid group than in the water group (Figure 9). Reductions in the mRNA expression of MuRF-1 and atrogin-1 were observed in the soleus muscle of the acetic acid group, as shown in Figure 3. The forkhead box protein O (FOXO) transcription factor promotes muscle atrophy through the induction of MuRF-1 and atrogin-1. The phosphorylation and inhibition of FOXO is mediated by phosphorylated Akt [36,37,38,39]. We examined the phosphorylation of Akt and FOXO in the soleus muscle of rats in the acetic acid group. The phosphorylation levels of Akt, FOXO1, and FOXO3a were significantly higher and the phosphorylation ratios of AMPK, Akt, and FOXO1 were also significantly increased in the soleus muscle of rats in the acetic acid group than those in the water group. The level of phosphorylated Ser-2448 mTOR did not differ between the two groups (data not shown). These proteins did not change in the gastrocnemius muscle between the two groups (data not shown). 

## 3. Discussion

Aging contributes to a decline in the oxidative capacity of muscles [9]. Our previous study showed that VO_2_ during both active and resting periods decreases significantly with age [40]. In this study, the VO_2_ in the acetic acid group did not decrease significantly during the experimental period from 36 to 55 weeks of age. Thus, long-term supplementation with acetic acid could attenuate age-related decline in O_2_ consumption and energy metabolism. 

The number of type I fiber in soleus muscle of the water group at 56 weeks of age was significantly decreased as compared to rats at 20 weeks of age. However, the number of type I fibers in the soleus muscles of the rats were significantly increased in the acetic acid group compared with that in the water group at 56 weeks of age (Figure 2B). The number of type II fibers in the soleus and gastrocnemius muscles was significantly decreased in the rats in the water and acetic acid groups at 56 weeks of age as compared with that in rats aged 20 weeks (Figure 2B,C). Age-associated muscle atrophy is due in part to a decrease in muscle fiber numbers [41,42]. Long-term supplementation with acetic acid protected against the decrease in the number of type I fibers in the soleus muscles of rats. Previous studies have suggested that the loss of muscle mass with unloading occurs primarily through an increase in the degradation of myofibrillar proteins via the ubiquitin–proteasome pathway [43,44,45]. Long-term supplementation with acetic acid suppressed the mRNA expression of MuRF1 and TGF-β in the soleus and gastrocnemius muscles (Figure 3). TGF-β inhibits myogenesis and prevents the activation of transcriptional complexes related to MEF2A through the cytoplasmic localization of MEF2A [46]. MEF2A is a transcription factor involved in skeletal muscle differentiation and is associated with the stimulation of type I fiber proteins [34]. MEF2A expression was higher in the soleus muscles of the rats in the acetic acid group than in those of the rats in the water group (Figure 4A). The levels of myoglobin and troponin I genes, which are associated with type I fibers, were significantly increased in the gastrocnemius muscles of the rats in the acetic acid group compared with those in the rats in the water group (Figure 4B). In the soleus muscles of the rats in the acetic acid group, MEF2A and PGC-1α protein levels showed an increase, and the nuclear localization of MEF2A and PGC-1α was also increased in the acetic acid group compared with that in the water group (Figure 5A,C). In the gastrocnemius muscle, the levels of PGC-1α increased in the nuclear fraction (Figure 5D). PGC-1α plays a key role in the regulation of mitochondrial biogenesis and oxidative metabolism. PGC-1α protects skeletal muscle from atrophy by suppressing FOXO3 action [47]. Increased muscle PGC-1α expression protects skeletal muscle from sarcopenia and metabolic diseases during aging [48]. PGC-1α drives the formation of slow-twitch fibers [49]. The promoter sequence of PGC-1α contains an MEF2 binding site and is regulated by mutual interactions [50]. The activities of PGC-1α and MEF2A are regulated by AMPK, which is the master regulator of metabolic homeostasis [51]. This regulation leads to increased mitochondrial biogenesis in skeletal muscles [22]. In L6 cells, acetic acid activates AMPK, leading to increased MEF2A and PGC-1α levels, and induces the expression of genes related to slow-twitch fibers and mtDNA [17].

SDH staining characterizes mitochondrial enzyme function and the oxidative capacity of myofibers [52,53,54]. We observed that SDH staining levels were significantly increased in the soleus and gastrocnemius muscles of the rats in the acetic acid group (Figure 6B,C). The levels of mtDNA also increased in the soleus muscles of the acetic acid group rats (Figure 6A). GPR43 expression was stimulated by acetic acid treatment in the soleus muscles of the rats. In our previous study, the treatment of L6 myotube cells with acetic acid induced the expression of several genes associated with slow-twitch fibers, such as those encoding MEF2A, myoglobin, PGC-1α, and SDH, through the activation of AMPK and GPR43 [16,17]. Acetic acid functions in the activation of GPR43 and the induction of calcium influx, leading to the proliferation of slow-twitch fibers in L6 cells [17]. The results of the present study indicate that long-term supplementation with acetic acid affects the proliferation of type I fibers in the soleus muscles and type I fiber-associated genes in the soleus and gastrocnemius muscles of aging rats. They also indicate mitochondrial proliferation in these muscles after long-term supplementation with acetic acid. Efficient skeletal muscle bioenergetics largely rely on the metabolic flexibility of the mitochondria [3,4]. Mitochondrial function in skeletal muscle declines with aging, concomitant with decreased exercise and physical activity [5,6]. Aged muscle fibers have an impaired capacity to oxidize metabolic fuels in the mitochondria. Previous studies have indicated that aged muscle reduces skeletal muscle mitochondrial biogenesis, the expression of mitochondrial respiratory complex subunits, mitochondrial respiration, and ATP levels [8,55]. Long-term supplementation with acetic acid protected against lipid accumulation, necrosis, and myofibrosis of the soleus muscle (Figure 8). In soleus muscle, phosphorylated AMPK levels were significantly increased following acetic acid treatment. AMPK activation is linked to lipid catabolism, the improvement of insulin sensitivity [19], and the modulation of mitochondrial oxidative capacity [17,23].

Phosphorylated Akt was also activated and showed an increase in its levels, whereas FOXO1 and FOXO3a were phosphorylated and inactivated in the soleus muscle (Figure 9). Akt protects against the expression of atrophy-related ubiquitin ligases, MuRF-1 and atrogin-1, through the suppression FOXO activity [36,37,38,39]. These findings suggest that acetic acid supplementation activates both the AMPK and Akt signaling pathways. An active ingredient of ginsenosides, G-Rk3, reportedly activates the AMPK/Akt signaling pathway in high-fat diet/type 2 diabetes-induced mice [56]. G-Rk3 suppresses high-fat diet- and streptozotocin-induced lipid accumulation by regulating proteins downstream of AMPK/Akt. In addition, biglycan improves glucose metabolism through the AMPK/AKT pathway in skeletal muscle [57].

In conclusion, the collective results of the present study indicate that long-term supplementation with acetic acid may attenuate the age-related decline in mitochondrial function and improve lipid deposition in skeletal muscle. Acetic acid would protect against metabolic diseases associated with the dysregulation of energy metabolism during the aging process. Further explorations are required before acetic acid supplementation can be applied clinically.

## 4. Materials and Methods

### 4.1. Materials

Sepasol-RNA I Super G was purchased from Nacalai Tesque (Kyoto, Japan). Isopentane, formaldehyde, 2-mercaptoethanol, and 1% eosin Y solution were purchased from FUJIFILM Wako Pure Chemical Corporation (Osaka, Japan). The PrimeScript RT Reagent Kit with gDNA Eraser was purchased from TaKaRa Bio (Shiga, Japan). SYBR FAST qPCR kits were purchased from Kapa Biosystems (Wilmington, MA, USA). Primary antibodies against AMPKα (#2532), phosphorylated Thr-172 AMPKα (#2535), Akt (#9272), phosphorylated Ser-473 Akt (#4058), forkhead box protein O1 (FOXO1) (#2880), phosphorylated Ser-256 FOXO1 (#9461), FOXO3a (#12829), and phosphorylated Ser-253 FOXO3a (#13129) were purchased from Cell Signaling Technology (Danvers, MA, USA); MEF2A (sc-313), PGC-1α (sc-517380), and Sp1 (sc-14027) were purchased from Santa Cruz Biotechnology (Dallas, TX, USA); and α-tubulin (#017-25031) was obtained from FUJIFILM Wako Pure Chemical Corporation (Osaka, Japan). For secondary antibodies, goat anti-mouse IgG H&L (HRP) (ab6789) and goat anti-rabbit IgG H&L (HRP) (ab6721) were purchased from Abcam plc. (Cambridge, UK), and goat anti-rabbit IgG-HRP (sc-2004) was purchased from Santa Cruz Biotechnology. Polyvinylidene difluoride membrane (Immobilon-P, 0.45μm) for Western blotting was purchased from Merck (Darmstadt, Germany).

### 4.2. Animal Experiments

All animal experiments were conducted in accordance with the guidelines of Okayama Prefectural University. The care and use of the animals in this study followed the guidelines of the Okayama Prefectural University and the laws and notifications of the Japanese government. All animal experiments were approved by the Animal Care and Use Committee of the Okayama Prefectural University (protocol number 27-3). Thirty-two-week-old male SD rats were used in this study. They were fed a normal laboratory diet (CE-2, CLEA Japan, Inc., Tokyo, Japan) for 4 weeks to stabilize their metabolic conditions. The rats were housed individually in an air-conditioned room at approximately 25 °C with alternating 12 h periods of light and dark conditions (light during 08:00–20:00). All animals were allowed free access to water and the laboratory diet. The rats were randomly assigned to two groups: water-injected and acetic acid-injected. The water group was administered orally distilled water at 5 mL/kg body weight, and the acetic acid-injected group was administered orally 1% (*v*/*v*) acetic acid at 5 mL/kg BW daily 5 days a week from 37 to 56 weeks of age, although 56 weeks of age is not considered as old age in SD rats. Final body weight at 56 weeks of age (water group; 714 ± 30.0 g, acetic acid group; 673 ± 9.17 g, *p* = 0.285) and total food intake (water group; 3.5 ± 0.14 kg, acetic acid group; 3.3 ± 0.05 kg, *p* = 0.324) during the feeding period were not significantly different between the two groups. As a younger control group, rats at 8 weeks of age were injected with distilled water until 20 weeks of age. 

VO_2_ was measured using an O_2_/CO_2_ metabolism measuring system (MK-5000; Muromachi Kikai, Tokyo, Japan). This system monitors VO_2_ and VCO2 at 3 min intervals. Each rat was placed in a sealed chamber with a constant air flow (3.0 L/min) for 24 h at 25 °C with free access to water and food. Measurements were performed during the dark or light periods. 

Food consumption and BW were recorded daily. At 56 weeks of age, the rats were anesthetized by intraperitoneal injection of nembutal (Sumitomo Dainippon Pharma, Tokyo, Japan). Tissue samples were collected in 24 h after the final administration of water or acetic acid in the fed state. The soleus and gastrocnemius muscles were immediately isolated, frozen in liquid nitrogen, and stored at −80 °C for RT-PCR, mitochondrial DNA, and Western blotting analyses.

### 4.3. Histological Analysis

The skeletal muscles were quickly frozen in isopentane chilled with liquid nitrogen, and then they were frozen in liquid nitrogen. Cryostat sections 10 μm thick were obtained at −20 °C and fixed in 10% Formalin Neutral Buffer Solution (FUJIFILM Wako Pure Chemical Corporation, Osaka, Japan). The sections were stained with hematoxylin and eosin (HE), and mounted with Mount-Quick (Daido Sangyo Co., Ltd., Saitama, Japan) mounting medium. For Oil Red O staining, the sections were fixed in 10% Formalin Neutral Buffer Solution, incubated in 60% isopropanol, and stained with Oil Red O staining solution for 15 min at 20–25 °C. This was followed by washing briefly with 60% isopropanol and tap water, and then stained by hematoxylin for 3 min, washed with distilled water, and mounted with Mount-Quick AQUEOUS (Daido Sangyo Co., Ltd., Saitama, Japan) mounting medium. For adenosine triphosphatase (ATPase) staining, unfixed sections were pre-incubated at 20–25 °C for 10 min in a buffer consisting of 100 mM potassium chloride in 100 mM sodium acetate, adjusted to pH 4.5 with acetic acid. Sections were washed for 30 s in a 20 mM glycine buffer (pH 9.45) containing 20 mM CaCl_2_. This was followed by 1 h of incubation at 20–25 °C room temperature in 40 mM glycine buffer (pH 9.45) containing 20 mM CaCl_2_ and 2.5 mM ATP disodium salt. The sections were washed using three 30 s changes of 1% CaCl_2_ and then were kept in 2% CoCl_2_ for 3 min. This was followed by three 30 s changes of distilled water, immersion in 1% yellow ammonium sulfide for 5 min, washing in distilled water, and embedding in glycerol jelly. For SDH staining, the sections were incubated with 50 mM phosphate buffer (6 mM potassium dihydrogen phosphate and 44 mM disodium hydrogen phosphate), 50 mM succinate, and 0.5 mg/mL nitroblue tetrazolium for 40 min at 37 °C. The sections were briefly washed three times with distilled water and embedded in glycerol jelly. Images were captured using a CCD camera (Olympus Optical, Tokyo, Japan) at a magnification of x100.

### 4.4. Quantitative RT-PCR Analysis

Total RNA was isolated from frozen tissue samples with Sepasol-RNA I super G. Genomic DNA was isolated using extraction buffer (4 M guanidine thiocyanate, 50 mM sodium citrate, and 1 M Tris). An RNase inhibitor was added according to the manufacturer’s instructions. Total RNA was quantified, and cDNA was prepared using the PrimeScript® RT Reagent Kit with gDNA Eraser (TaKaRa Bio, Shiga, Japan) according to the manufacturer’s instructions. Quantitative real-time PCR analyses were performed using the StepOnePlus detection system (Applied Biosystems, Foster City, CA, USA) with KAPA SYBR FAST qPCR kits to quantify the specific mRNA content. Data were normalized to β-actin mRNA levels and expressed relative to the control group. The oligonucleotide primer sequences used in this study are listed in Table 1. 

### 4.5. Nuclear Extraction and Western Blotting

Frozen tissue was homogenized in extraction buffer (25 mM Tris HCl (pH 8.0), 1 mM EDTA, 0.5 mM dithiothreitol (DTT), 10 mM MgCl_2_, 0.25 mM sucrose, 50 mM sodium fluoride, and protease inhibitor) and centrifuged at 3000 rpm for 10 min at 4 °C. The supernatant was used as the extract from total fraction. To extract cytosolic and nuclear fractions, frozen tissue was cut into small pieces, homogenized in lysis buffer (20 mM HEPES pH7.9, 3 mM MgCl_2_, 20 mM KCl, 1 mM DTT, and protease inhibitors) with glass homogenizer, centrifuged at 10,000× *g* for 20 min at 4 °C, and the supernatant was collected as the extract from cytosolic fraction. The pellet was washed with ice-cold PBS, resuspended in washing buffer (20 mM HEPES pH7.9, 1.5 mM MgCl_2_, 0.6 mM EDTA, 0.42 M NaCl, 25% (*v*/*v*) glycerol, 1 mM DTT, and protease inhibitors) by pipetting gently, and centrifuged at 8500× *g* for 5 min at 4 °C. Then, the pellet was collected, resuspended in extraction buffer (0.32M sucrose, 1mM MgCl_2_, and 1mM potassium phosphate) with an ultrasonicator, shaken at 4 °C for 30 min, centrifuged at 20,000× *g* for 10 min at 4 °C, and the supernatant was collected as the extract from the nuclear fraction. After the separation of the cytosolic and nuclear fractions, the protein concentration of each extract from nuclear, cytosolic, and total fractions were determined by the Bradford assay. An aliquot (15–30 μg of protein) of each extract was used for Western blotting to determine the protein content of AMPKα, phosphorylated Thr-172 AMPKα, MEF2A, PGC-1α, Akt, phosphorylated Ser-473 Akt, FOXO1, phosphorylated Ser-256 FOXO1, FOXO3a, phosphorylated Ser-253 FOXO3a, Sp1, and α-tubulin. Samples were resolved by 7.5–10% SDS-PAGE, and then proteins on the gel were transferred onto a polyvinylidene difluoride membrane. The membrane was first incubated with primary antibody overnight at 4 °C, washed three times with TBST, and incubated with HRP-conjugated secondary antibody for 60 min. After washing three times with TBST, the chemiluminescent reaction was performed for 5 min with ImmunoStar LD (FUJIFILM Wako Pure Chemical Corporation, Osaka, Japan), according to the protocol supplied by the manufacturer. Chemiluminescent signals were visualized and quantified with ImageQuant LAS-4000 and Multi Gauge V3.2 analyzing software (Fujifilm, Tokyo, Japan).

### 4.6. Mitochondrial DNA Analysis

Genomic DNA was extracted from the soleus and gastrocnemius muscles of rats administered water or acetic acid. The content of mtDNA was analyzed by measuring the relative copy number of the mitochondrial encoded gene, NADH dehydrogenase subunit 1 (*mt-Nd1*), and nuclear DNA encoded gene, β-actin (*Actb)*, by quantitative real-time RT-PCR.

### 4.7. Statistical Analyses

Data are presented as mean ± SE. Results were analyzed using one-way ANOVA, followed by the Tukey–Kramer post hoc test for multiple comparisons or analyzed with unpaired Student’s *t*-test for direct comparison between two groups. Statistical significance was set at *p* < 0.05. All statistical analyses were performed using statistical database software (SPSS Statistics 27.0 software for Microsoft Windows, IBM, Chicago, IL, USA).

## Figures and Tables

**Table 1:** List of sequences of PCR primers.

Gene	Forward	Reverse
β-actin (*Actb*)	GGAGATTACTGCCCTGGCTCCTA	GACTCATCGTACTCCTGCTTGCTG
Atrogin-1 (*Fbxo32*)	TCCAGACCCTCTACACATCCTT	CCTCTGCATGATGTTCAGTTGT
MuRF1 (*Trim63*)	TGCATCTCCATGCTGGTGGC	CTTCTTCTCGTCCAGGATGG
TGF-β (*Tgfb1*)	ATAGCAACAATTCCTGGCGTTACC	CACTGAAGCGAAAGCCCTGTATTT
MHC1 (*Myh7*)	AGAGGAAGACAGGAAGAACCTAC	GGCTTCACAGGCATCCTTAG
MEF2A (*Mef2a*)	ATGAGAGGAACCGACAGGTG	TATCCGAGTTCGTCCTGCTT
Myoglobin (*Mb*)	CTAACAGCCGGCCTACACTC	CGTGCTTCTTCAGGTCCTCT
Toroponin I (*Tnni1*)	CGAGCCCTACTGGGTTCCAA	CAGACATGGCCTCCACGTTC
PGC-1α (*Ppargc1a*)	GACCCCAGAGTCACCAAATGA	GGCCTGCAGTTCCAGAGAGT
Succinate dehydrogenase (*Sdha*)	TGGGGCGACTCGTGGCTTTC	CCCCGCCTGCACCTACAACC
GLUT4 (*Slc2a4*)	GGGCGATTTCTCCCACATAC	CTCATGGGCCTAGCCAATG
GPR43 (*Ffar2*)	CAGAGGAGAACCAGGTGGAAG	GGCAGGGACCCCAGTAAGAA
NADH dehydrogenase 1, mitochondrial (*mt-Nd1*)	CTCCCTATTCGGAGCCCTAC	ATTTGTTTCTGCTAGGGTTG

## Data Availability

Not applicable.
